# **Immune risk assessment of residual** α**Gal in xenogeneic decellularized cornea using GTKO mice**

**DOI:** 10.1093/rb/rbaa020

**Published:** 2020-06-23

**Authors:** Liang Chen, Lina Wei, Anliang Shao, Liming Xu

**Affiliations:** Department of Medical Device, National Institutes for Food and Drug Control, Huatuo Road 31, Biomedical Production Zone, Daxing District, 102629 Beijing, China

**Keywords:** decellularized corneal matrix, immune risk, galactose-α1,3-galactose (αGal), GTKO mice

## Abstract

The xenogeneic decellularized corneal matrix (DCM) was expected to be used in lamellar keratoplasty in clinic as the substitute of allogeneic cornea. After decellularization treatment, the remaining risk of xenograft rejection needed to be assessed. The galactose-α1,3-galactose, as the most abundant and closely rejection-related xenogeneic antigen, should be one of the important factors concerned in immunological evaluation. In this study, residual αGal in the DCM was first determined by an enzyme-linked immunosorbent assay method with qualified accuracy and specificity. Then the DCM was implanted subcutaneously into the α1,3-galactosyltransferase gene-knockout (GTKO) mice, accompanied by the implantation in the wild-type C57BL/6 mice as a comparison. The total serum antibody levels, anti-Gal antibody levels, inflammatory cytokines and ratios of splenic lymphocyte subtypes were detected and the histopathological analysis of implants were performed to systematically evaluate the immune responses. The experimental result showed the fresh porcine corneal matrix samples had (9.90 ± 1.54) × 10^12^ αGal epitope per mg while the content of residual αGal in the DCM was (7.90 ± 2.00) × 10^12^ epitope per mg. The GTKO mice had similar potential of reaction to immune stimulation to that of wild-type C57BL/6 mice. At 4 weeks after implantation of DCM, in WT mice and GTKO mice there were both innate immunity response to the DCM characterized by macrophage infiltration. But the elevations of anti-Gal IgG level and the percentage of splenic natural killer cells were only detected in GTKO mice. These changes were thought to be pertinent to the residual αGal antigen, which could not be detected in WT mice. No further αGal antibody-mediated cellular immunity and significant changes of serum cytokine contents were found in GTKO mice, which perhaps suggested that the immune reactions to the DCM after 4 weeks of implantation were moderate and had minor effect on the survival of the corneal graft.

## Introduction

Corneal transplantation as the ultimate therapy to avoid blindness due to corneal diseases had great clinical needs. However, due to the lack of allogeneic cornea, heterokeratoplasty had attracted much attention as an alternative. Since galactose-α1,3-galactose (αGal) existed in large quantity on the surface of xenogeneic corneal cells after transplantation, to which the human immune system was highly responsive, the immune rejection of xenografts was the major obstacle to graft survival. The researchers tried different strategies and methods to improve the functional survival of xenografts in the recipient.

The decellularization treatment could reduce the immunogenicity associated with xenogeneic cells. There were many ways of corneal decellularization processing, including freeze drying [1], hypoxic nitrogen [2], hypertonic saline [3], treatment with sodium dodecyl sulfate (SDS) [4], treatment with phospholipase A2/sodium deoxycholate [5] and supercritical carbon dioxide extraction technology [6]. Porcine cornea had a tissue structure similar to that of human cornea, and the porcine DCM might be used in lamellar keratoplasty where the recipient cornea’s innermost layer of corneal endothelial cells was intact. The DCM was expected to be used as scaffold material in which corneal cells could regenerate. It had been found that the decellularized corneal had a longer survival time than fresh corneal graft [7].

Immune privilege existed in normal cornea due to lack of vascular structure [8]. However, if corneal transplantation was needed clinically, there might be infection, inflammation and trauma in the affected area of cornea, which could lead to angiogenesis [9]. When decellularized corneal graft was transplanted into a vascularized cornea, the immune cells of the human body would come into contact with the xenogeneic tissue. On the other hand, in order to control the effect of decellularization treatment on corneal tissue structure and mechanical properties, it was impractical and impossible to completely eliminate heterologous antigens in the cornea. Therefore, the immune responses to the decellularized cornea that affected the success of the transplantation needed to be evaluated.

In the corneal stromal capsule implantation or lamellar corneal transplantation, some experimental results showed there were no evidence of inflammatory cells infiltration, corneal neovascularization and rejection occurred in the porcine DCM treated with hypertonic saline or SDS solution [3, 4]. But in the other experiments, there were reported that inflammatory cells infiltration, corneal neovascularization and rejection had occurred in the porcine DCM treated with freeze drying [1], hyperosmotic glycerol [3], SDS solution [10] and Triton X-100 [11]. It was worth noting that even there was an immunocyte infiltration in the porcine DCM treated with hypertonic saline/Triton X-100 after implantation in the rabbit corneal stromal capsule, but when the same porcine DCM was used in rabbit lamellar corneal transplantation in which the experimental corneal ulcer had been produced before, there were obviously immunocyte infiltration and rejection occurred which led to degradation of the porcine DCM [12]. These inconsistent results suggested the need for immunological evaluation of decellularized cornea, especially considering there were various kinds of decellularization treatments.

Although xenogeneic rejection was associated with many heterologous antigens [13], αGal, as the most abundant and closely rejection-related xenogeneic antigen, should be one of the important factors concerned in immunological evaluation [14]. In view of animals adopted in the above experiments, both rat and rabbit were species expressing α-Gal antigen, which could not respond to the remnant αGal in decellularized cornea. Non-human primate, which did not express αGal, was a suitable animal model that could reflect the immune response to αGal antigen due to the existence of high level of anti-Gal antibody. But the use of this large animal model was strictly limited.

Transgenic animals which did not express αGal antigen could be obtained from wild animals by α1,3-galactosyltransferase gene-knockout (GTKO) technique [15]. The GTKO animals preimmunized with αGal antigen could produce similar level of anti-Gal antibody to that of human [16]. Therefore, it was a reasonable choice to evaluate the immune response to xenogeneic decellularized cornea in GTKO animals. But at present, the immunological evaluation of decellularized cornea carried out in GTKO animals was very little. In the experiment of porcine DCM orthotopic transplantation, there was found no increase in the anti-Gal antibody level in sensitized GTKO mice [17]. But the limiting factors of the study needed to be considered. The researchers pointed out that the mouse cornea was too thin to separate the corneal endothelial cell layer and porcine DCM was transplanted to the whole corneal defect. But the graft was edema and opacity on the second day because the water was not discharged from the DCM without endothelial cell layer. This seemed to suggest that GTKO mice was unsuitable for *in situ* evaluation of decellularized cornea.

Therefore, in this study, we carried out the subcutaneous implantation of decellularized cornea in GTKO mice to obtain immune response information, as the subcutaneous implantation had been adopted in the immunological evaluation experiments of other xenografts such as the decellularized lung scaffolds [18] and the bovine pericardia [19]. We also carried out the immunological evaluation of decellularized cornea in wild-type C57BL/6 mice for the comparison between animal models. The relationship between the subcutaneous implantation of decellularized cornea and the clinical application was weaker than that of *in situ* implantation, but because the subcutaneous environment was more vulnerable to neovascularization, it might mimic the conditions of pathological vascularized corneal bed found in clinical, which could be seen as the worst case of immune hazard assessment.

## Materials and methods

### Preparation of DCM

Pig eyes were obtained from the local slaughterhouse, placed in phosphate-buffered saline (PBS 0.1 M, pH 7.4) and immediately transported to our laboratory. The eyes were thoroughly washed with carbonate buffer (pH 8.3) to clean the corneas and the corneas with diameter 10 mm were extracted by corneal trephine. At first, corneas were soaked in ultrapure water to allow swelling for 12 h. Then Corneas were immersed in decellularization solution I (carbonate buffer containing 0.5% sodium deoxycholate and 200 U/ml phospholipase A2) for 6 h. After washed in carbonate buffer solution, the corneas were immersed in decellularization solution II (carbonate buffer containing 200 U/ml phospholipase A2) for 2 h and then washed in the carbonate buffer again. Finally, the decellularized corneal matrices were dehydrated and packed and the sterilization was performed by ^60^Co irradiation.

### Determination of αGal antigen in corneal matrix by ELISA

The αGal contents of fresh corneal matrix and DCM were quantitatively detected by an inhibitory enzyme-linked immunosorbent assay (ELISA) [20]. Firstly, the lysates of corneal matrix were prepared by homogenized in lysis buffer containing 1% protease inhibitor PMSF using a homogenizer (Benchmark D1000-E, USA), incubating at room temperature for 1–3 h, and making sure that there were no obvious solid matters and the α-Gal antigen was completely exposed. An αGal antigen quantitative detection kit was adopted (MeiTan 70101, Beijing SaoYao, China). The Gal-α1, 3Gal-BSA (Gal-BSA) (NGP0203, Dextra Laboratories, UK) in the kit was used as the standard for quantitative analysis, of which the amount of αGal was 1.82 × 10^17^ epitope/mg. The ELISA was performed by following the instruction. Briefly, the lysates of corneal matrix and the Gal-BSA standard solution were incubated with the monoclonal antibody M86. Then the residual M86 antibody was loaded into the Gal-BSA pre-coated 96-well plate followed by the enzymatic chromogenic reaction of horseradish peroxidase (HRP)-conjugated secondary antibody. The inhibitory degree of the corneal matrix to the chromogenic reaction was inversely proportional to the amount of αGal epitope. Therefore, the content of the αGal in the corneal matrix could be accurately calculated from the Gal-BSA standard curve.

### Animals and surgical procedure

5–6-week-old GTKO female mice and wild-type (WT) C57BL/6 female mice were obtained from the Institute for Laboratory Animal Resources of National Institutes for Food and Drug Control (NIFDC, China). The study protocol was approved by the NIFDC Institutional Animal Care and Use Committee. The GTKO mice were preimmunized once every 2 weeks by intraperitoneal injection of rabbit red blood cell (RRBC), each time 1 × 10^8^ RRBC. The experiment was started at 1 week after the second immunization. The GTKO mice and WT mice were divided into two groups, respectively: blank control (CON) group and DCM group (*n* = 4 per group). Before transplantation, all animals were anesthetized by intraperitoneal injection of 5% chloral hydrate. All surgery was performed by using aseptic techniques. One piece of DCM with diameter 10 mm was implanted subcutaneously in the dorsal area of each GTKO mice and WT mice in DCM group, while mice in control group received sham surgery. At 4 weeks after implantation, blood was obtained from anesthetized animals of all groups by retro-orbital puncture for the determination of antibodies and cytokines. After the mice were killed by dislocation of cervical vertebra, tissue samples of spleen, thymus and implants were also harvested for immune reaction test and pathological analysis.

### Measurement of serum IgG and IgM antibodies

At 4 weeks after implantation, serum was collected from WT mice and GTKO mice. The total IgG and IgM antibodies in WT and GTKO mice serum were measured by ELISA kits (IgG, EMC116, Neobioscience, China; IgM, 88-50470-22, affymetrix Thermo Fisher scientific, USA), following the manufacturer’s instructions.

### Evaluation of serum anti-Gal IgG and IgM antibodies

Serum was collected from WT mice and GTKO mice at 4 weeks after implantation and stored at −80°C until use. The serum anti-Gal IgG and IgM antibodies were measured by ELISA. Briefly, the plates were coated with 100 μL Gal-BSA (2 μg/ml, NGP0203, Dextra Laboratories, UK) and incubated overnight at 4°C. After blocking with 1% human serum albumin (A8230-1, Sigma), serial dilutions of serum (IgG 1:50–1:200, IgM 1:100–1:400) were added into each well and incubated at 37°C for 2 h, followed by incubation with the HRP-conjugated IgG or IgM antibodies (sc-2005, Santa Cruz Biotechnology). The absorbance was then measured at 450 nm using a microplate reader (SpectramaxM5, Molecular Devices, USA).

### Assay of cytokine concentrations in serum

The cytokine levels of IFN-γ, IL-4 and IL-12p70 in the serum of WT mice and GTKO mice at 4 weeks after implantation were tested by cytometric bead array analysis (CBA, BD Biosciences, USA). The CBA assay was performed according to the manufacturer’s instructions by flow cytometry (FACSCanto II, BD Biosciences) as previously described [21]. The concentration of cytokines was measured by FCAP Array^TM^ software.

### Detection of splenic lymphocyte surface molecules

Spleen was carefully dissected out after mouse was killed at 4 weeks. To prepare the splenic mononuclear cells, spleen tissue was minced, homogenized and filtered by 70 μm cell filter. After lysing the red blood cells, the mononuclear cells were centrifuged and resuspended with 1640 cell culture medium. For T lymphocytes detecting, cells were incubated with anti-CD45, anti-CD3, anti-CD4 and anti-CD8 antibodies in the dark at room temperature for 20 min, and for B lymphocytes detecting, cells were incubated with anti-CD45, anti-CD3, anti-CD19 and anti-CD69 antibodies in the dark at room temperature for 20 min. For detecting the natural killer (NK) cells and natural killer T (NKT) cells, the mononuclear cells were incubated with anti-CD45, anti-CD3, anti-CD49b and anti-CD69 antibodies in the dark at room temperature for 20 min. After two washes with 1% BSA-PBS, the cells were re-suspended in PBS with 1% BSA and were subjected to flow cytometry analysis (FACSCanto II, BD Biosciences).

### HE staining and immunohistochemistry

The samples of subcutaneous tissue surrounding the implant of each mouse were fixed in 10% buffered formalin solution for 48 h before paraffin embedding. Paraffin blocks were sectioned and stained with hematoxylin and eosin (H&E) for histological assessment. To evaluate for the presence of CD3^+^, CD4^+^, CD8^+^ cells and macrophages in the corneas, the following antibodies were used in immunohistochemistry: CD3e (Thermo fisher, Waltham, USA; MA1-90582), CD4 (Abcam, Cambridge, MA, USA; ab125212), CD8 (Thermo fisher MA5-14548), CD68 (Abcam ab125212). PBS was used as a negative control. The sections were examined under a light microscope (Leica, Wetzlar, Germany).

### Statistical analysis

Data were expressed as the mean ± SD. Effects of animal species and corneal implant on the determined immune indexes were compared by two-way ANOVA analysis with paired comparison adopting Bonferroni test. The values of anti-Gal IgG and IgM antibodies in GTKO mice were compared using the non-parametric Kruskal–Wallis test. Statistical significance was accepted for *P* values of < 0.05.

## Results

### Determination of αGal antigen in corneal matrix

After the homogenization and digestion, the content of αGal in corneal matrix was determined by ELISA. It was found that the fresh porcine corneal matrix samples (triplicate) had (9.90 ± 1.54) × 10^12^ αGal epitope per mg. The αGal content of DCM (triplicate) was decreased to (7.90 ± 2.00) × 10^12^ epitope per mg, which meant the clearance rate of αGal provided by decellularization processes was 20.2%.

### Total serum IgG and IgM contents

After 4 weeks of implantation, the total serum IgG and IgM of each mouse from the wild-type (WT) mice and GTKO mice were measured and the results were showed in Table 1. There was no statistically significant difference in serum total IgG and IgM content between WT mice and GTKO mice. Meanwhile, between the control group (CON) and DCM group there were no significant difference in total serum IgG and IgM content either.

**Table 1:** Total serum IgG and IgM content (μg/ml)

Group	IgG		IgM	
	WT	GTKO	WT	GTKO
CON	339.8 ± 153.7	350.3 ± 47.2	93.4 ± 82.3	101.3 ± 32.3
DCM	414.7± 114.9	468.3 ± 198.2	76.5 ± 29.4	117.8 ± 51.0

### Serum anti-Gal IgG and IgM levels

**Figure 1:**
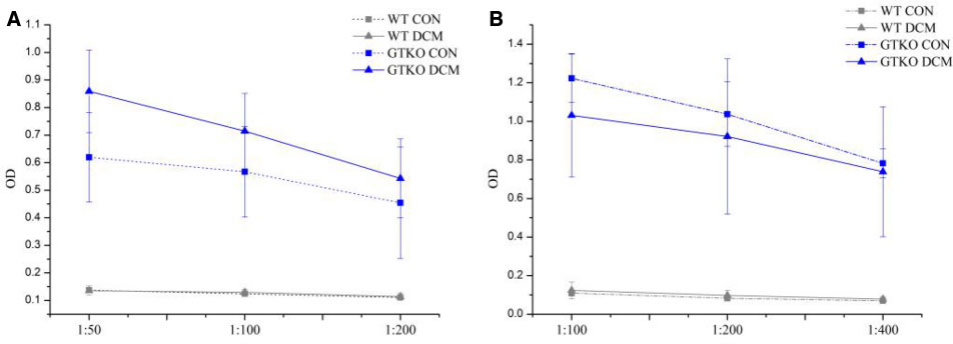
The serum anti-Gal IgG (**A**) and IgM (**B**) levels in the CON and DCM groups of WT and GTKO mice at 4 weeks after implantation. The values were represented as mean±SD.

The optical density (OD) values of serum anti-Gal IgG and IgM in both groups of the wild-type mice were very low (Fig. 1A and B). These were considered to be the background value of non-specific adsorption, which meant that no Gal antibody was produced in the WT mice. The control (CON) group of the GTKO mice received only sham-operation after RRBC (containing Gal antigen) preimmunization. Its test results indicated the existence of anti-Gal IgG and IgM, which showed that GTKO mice had reacted to the αGal antigen (Fig. 1A and B). In the GTKO mice, the absorbance values of serum anti-Gal IgG in the DCM group were higher than those of the CON group at all dilutions (Fig. 1A). And at the dilution of 1:50, the serum anti-Gal IgG level of DCM group of GTKO mice was significantly higher than that of the CON group (*P* < 0.05). On the other hand, the absorbance values of serum anti-Gal IgM in the DCM group of GTKO mice were lower than those of the CON group (Fig. 1B), but there was not the significant difference between two groups at the same dilution (*P* > 0.05).

### Serum cytokines

**Figure 2:**
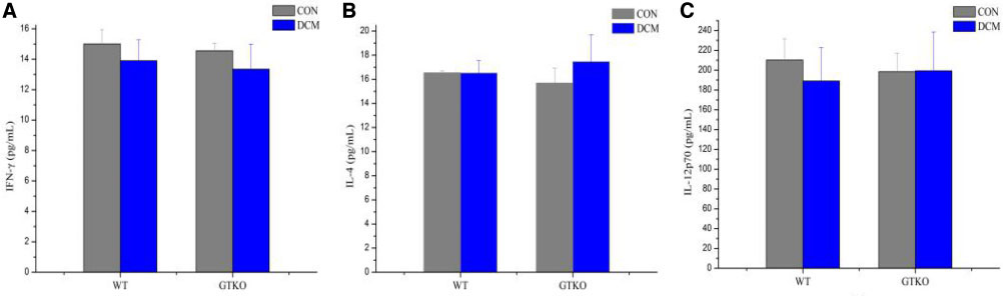
The serum cytokines level of IFN-γ (**A**), IL-4 (**B**) and IL-12p70 (**C**) in the control (CON) group and decelluarized corneal matrix (DCM) group of the WT and GTKO mice after 4 weeks of implantation. The values were represented as mean ± SD.

As shown in Fig. 2, the detected levels of IFN-γ, IL-4 and IL-12p70 showed no differences between the WT and GTKO mice. After 4 weeks of implantation of the DCM in the WT and GTKO mice, there were no significant changes in the serum cytokine levels of IFN-γ, IL-4 and IL-12p70 compared to the control groups. This indicated that the DCM did not stimulate the production of these cytokines both in the WT and GTKO mice.

### Ratios of T-lymphocytes, B-lymphocytes, and NKT-lymphocytes

**Figure 3:**
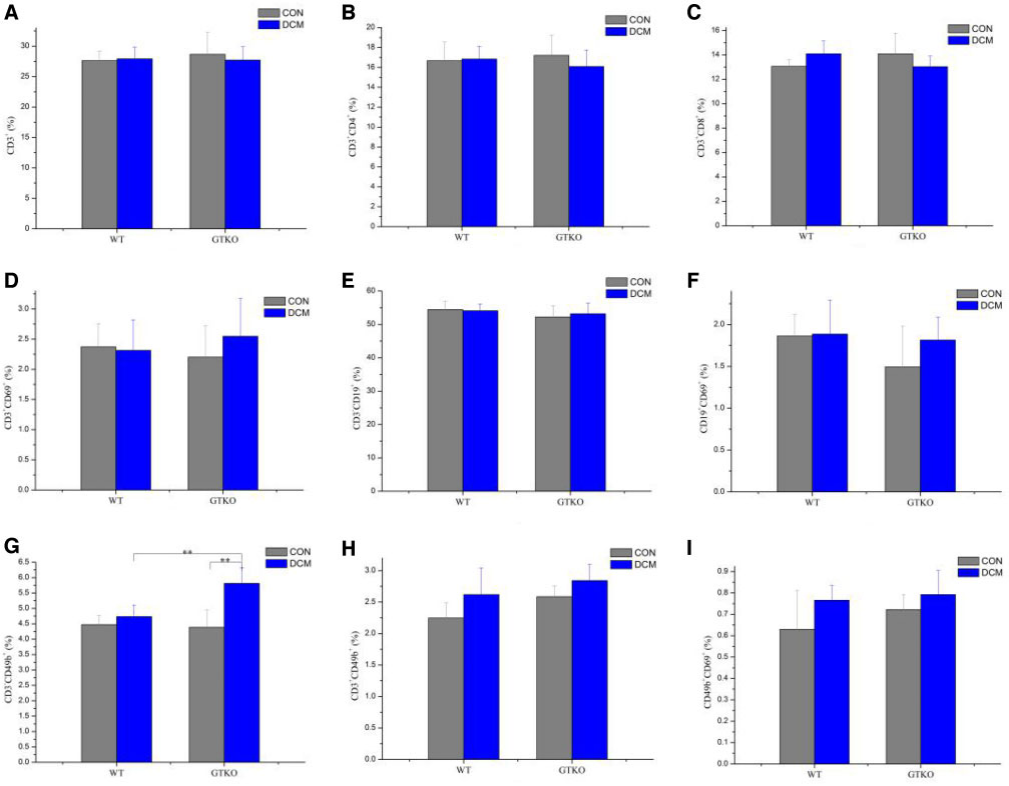
The ratios of CD3^+^ T-lymphocytes **(A**), CD3^+^CD4^+^ T-lymphocytes (**B**), CD3^+^CD8^+^ T-lymphocytes (**C**), CD3^+^CD69^+^ activated T-lymphocytes (**D**), CD3^-^CD19^+^ B-lymphocytes (**E**), CD19^+^CD69^+^ activated B-lymphocytes **(F**), CD3^-^CD49b^+^ NK cells (**G**), CD3^+^CD49b^+^ NKT (**H**) and CD49b^+^CD69^+^ activated NKT (**I**) in the control (CON) group and decelluarized corneal matrix (DCM) group of the WT and GTKO mice after 4 weeks of implantation, of which spleen single cell suspensions were analyzed by flow cytometry. The values were represented as mean ± SD. *P <0.05, **P < 0.01.

After the spleen single cell suspensions were labeled with various monoclonal antibodies, the ratios of T-lymphocytes, B-lymphocytes and NKT in the splenic lymphocytes were detected by flow cytometry. As shown in Fig. 3A–D, between the different mice (WT and GTKO mice) and between the different groups (CON and DCM groups), there were no significant differences in the ratios of CD3^+^, CD3^+^CD4^+^, CD3^+^CD8^+^ T-lymphocytes and activated T-lymphocytes marked by CD3^+^CD69^+^. The results showed the ratios of various T-lymphocytes in WT and GTKO mice were similar, and at 4 weeks after implantation, the DCM had not an effect on T-lymphocytes in both the WT and GTKO mice.

Moreover, the ratios of CD3^-^CD19^+^ B-lymphocytes and activated B-lymphocytes marked by CD19^+^CD69^+^, and ratios of CD3^+^CD49b^+^ NKT cells and CD49b^+^CD69^+^ activated NKT cells were both similar between the different groups and between the different animals (Fig. 3E, F, H and I). It indicated the ratios of these splenic lymphocytes in GTKO mice were comparable to those of the WT mice. And it showed that neither B-lymphocytes nor NKT cells were affected by the implantation of DCM in the WT and GTKO mice.

### Ratio of NK cells

After marked by monoclonal antibody of CD3 and CD49b, the expression of CD3^-^CD49b^+^ NK cells were observed by flow cytometry. As shown in Fig. 3G, the proportion of CD3^-^CD49b^+^ NK cells in GTKO control group was similar to that of WT control group. At 4 weeks after the implantation of DCM in the WT mice, the proportion of NK cells was not significantly different from that of the WT control group. However, the proportion of NK cells at 4 weeks in the GTKO DCM group was significantly higher than that of GTKO control group (*P* < 0.01), and the proportion of NK cells in DCM group of GTKO mice was also higher than that of the WT DCM group (*P* < 0.01). This indicated that in WT mice, no effect of the DCM on NK cells had been detected. But on the other hand, in the GTKO mice the stimulation of the DCM to NK cells was identified.

### Histopathology and immunohistochemistry

**Figure 4:**
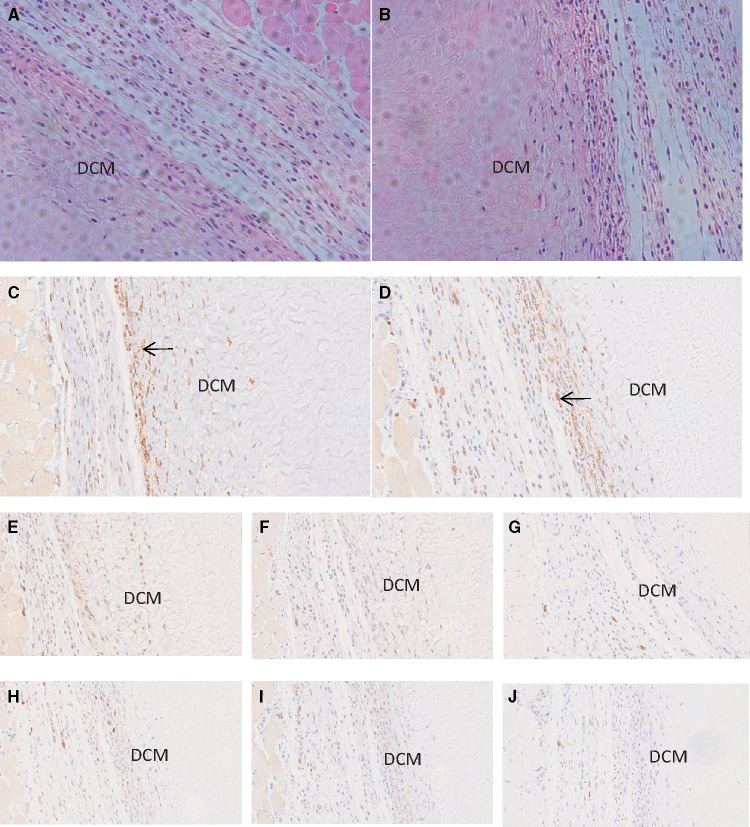
HE and immunohistochemical staining of infiltrating cells in decellularized corneal matrix after 4 weeks of implantation in the WT mice and GTKO mice. Figures of (**A**) HE staining, (**C**) CD68^+^, (**E**) CD3^+^, (**F**) CD4^+^, and (**G**) CD8^+^ were obtained from WT mice; (**B**) HE staining, (**D**) CD68^+^, (**H**) CD3^+^, (**I**) CD4^+^, and (**J**) CD8^+^ were images from GTKO mice. The black arrows showed the positive staining cells. DCM: decellularized corneal matrix.

In the HE staining sections of subcutaneous tissue of WT mice and GTKO mice, there were both found that collagen fibrous tissue were formed surrounding the implanted DCM, in which mononuclear cells were infiltrated. Moreover, some mononuclear cells had infiltrated into the adjacent portion of the DCM (Fig. 4A and B). Immunohistochemical staining of infiltrating cells in DCM showed many CD68^+^ macrophages were found (Fig. 4C and D), while CD3^+^, CD4^+^, CD8^+^ cells were barely seen (Fig. 4E–J). The type and number of infiltrating cells caused by the implantation of DCM were similar in WT mice and GTKO mice.

## Discussion

The contents of serum total IgG, total IgM and serum cytokines and percentages of spleen lymphocyte subtypes of GTKO control mice were not significantly different from those of WT control mice, suggesting that GTKO mice had similar potential of reaction to immune stimulation to that of WT mice. The increase of anti-Gal antibody level in GTKO mice after RRBC stimulation was in line with the expectation of preimmunization, which indicated that sensitized GTKO mice would response to αGal antigen exposed again.

After implantation of DCM in WT mice, there were no statistical differences between the blank control group and DCM group in the contents of total IgG, total IgM and serum cytokines (IFN- _γ_, IL-12p70 and IL-4), and the proportions of spleen lymphocyte subtypes. There were mononuclear cells infiltrating in or around the DCM as the HE sections showed, while immunohistochemical staining confirmed the vast majority of infiltrating cells were CD68^+^ macrophages, and CD4^+^ and CD8^+^ cells were barely seen. These results indicated in the WT mice the immune response to the DCM was mainly innate immune characterized by macrophage.

After implantation of DCM in GTKO mice, most of the measured items related to immune function did not change significantly compared with the blank control group. The local reaction to the DCM in the implantation site was innate immunity characterized by macrophage as shown by histopathological staining. These results suggested that the immunological responses of GTKO mice to implanted DCM were similar to those of WT mice, indicating that GTKO mice were as competent as WT mice in evaluating the immune response of xenogeneic DCM. On the other hand, after implantation of the DCM in GTKO mice, the changes of Gal antibody level and the percentage of spleen NK cell were observed in comparison with the blank control group. These two indexes might be related to αGal antigen. As the changes of these indexes did not occur in WT mice implanted with DCM, it might reflect the advantage of GTKO mice in evaluating xenogeneic DCM compared with WT mice.

The purpose of decellularization treatment was to reduce the contents of xenogeneic antigens. The αGal was the most abundant xenogeneic antigen and closely related to xenogeneic rejection. Therefore, the characterization of remnant content of αGal in decellularized cornea was of significance. The distribution of αGal in cornea had been characterized by immunofluorescence staining with *Griffonia simplifolia* I isolectin B4 (GSIB4), and the change of αGal content was explained by the change of fluorescence intensity [22]. But this was a qualitative or imprecise semi-quantitative method. An ELISA method of quantification of αGal in heart valve bioprostheses had been established [23]. In this method, using αGal contained in red blood cells as standard might have great variation. We have established an ELISA method using Gal-BSA as the standard, in which the αGal content of the current lot of Gal-BSA was precisely 1.82 × 10^17^ epitope/mg [24]. Furthermore, the liver tissues (without αGal antigen) of GTKO mice were included as negative control in the ELISA, which ensured the specificity and accuracy of the method [25]. It was found that the fresh porcine cornea samples had (9.90 ± 1.54) × 10^12^ αGal epitope per mg. The αGal content of DCM was decreased to (7.90 ± 2.00) × 10^12^ epitope per mg, which meant the clearance rate of αGal provided by decellularization processes was 20.2%. The phospholipase A2/sodium deoxycholate treatment in this experiment was similar to the literature [5], which indicated while maintaining the structure and mechanical strength of the cornea, the clearance of αGal was limited. So, the evaluation of immune effect of residual αGal was clearly warranted.

At 4 weeks after implantation of DCM into GTKO mice, the level of serum anti-Gal IgG was more than two times of that of the control group, and there was no significant difference of the anti-Gal IgM level between the DCM and the control group. These results showed that remnant αGal antigen in DCM could stimulate the production of IgG antibody in GTKO mice. Immune responses caused by residual αGal had also been reported in other xenogeneic decellularized tissues. When the decellularized heart valves were implanted subcutaneously in GTKO mice, there were found the increase of anti-Gal IgG and IgM antibodies. Moreover, macrophages and CD4^+^ T lymphocytes were found infiltrating in or around the subcutaneous implants in GTKO mice, while in WT mice CD4^+^ T lymphocytes were few or rare [26, 27]. These results indicated that αGal antibody-mediated cellular immunity had been induced by residual αGal. In this study, after implantation of DCM in WT and GTKO mice, the vast majority of inflammatory cells was macrophage, and both CD4^+^ and CD8^+^ T lymphocytes were barely seen, which meant no αGal antibody-mediated cellular immunity was found at 4 weeks after implantation of DCM into GTKO mice.

The reported immune effects induced by xenogeneic material implantation in GTKO mice also included the elevation of cytokine levels. Xenogeneic bone tissue had induced more than 10 times increase in anti-Gal IgG level of GTKO mice compared with the blank control, accompanied by a significant increase in serum IL-12p70 and IL-4 content [20]. However, at 4 weeks after implantation of the DCM, there were no significant differences in serum IFN-γ, IL-4 and IL-12p70 levels between the GTKO mice and the blank control group, although the elevation of the anti-Gal IgG antibody stimulated by residual αGal antigen contained in the DCM occurred.

After 4 weeks of implantation of the DCM, the percentage of splenic NT cells of GTKO mice was significantly higher than that of the blank control group. This difference might be also related to the residual αGal antigen. The NK cells were associated with the delayed xenograft rejection, in which the infiltrations of NK cells and macrophages were found [28, 29]. The inhibition of NK cell function was helpful to prolong the survival time of the xenograft [30]. The αGal antigen could directly stimulate NK cells to produce NK cell-mediated cytotoxicity and to secrete the IFN-γ [31, 32]. In this study, although the percentage of splenic NK cells increased after the DCM had been implanted into GTKO mice, the content of IFN-γ in serum was not significantly different from that of the blank control group, which meant the stimulating effect to NK cells by the residual αGal antigen was not so strong as to stimulate the secretion of IFN-γ.

## Conclusion

The GTKO mice had similar potential of reaction to immune stimulation to that of wild-type C57BL/6 mice which was reflected by the measured immune-related indexes. At 4 weeks after implantation of DCM, in WT mice and GTKO mice there were both innate immunity response to the DCM characterized by macrophage infiltration. Moreover, elevations of anti-Gal IgG level and the percentage of splenic NK cells were only detected in GTKO mice. These changes were thought to be pertinent to residual αGal antigen which could not be induced in WT mice. No further αGal antibody-mediated cellular immunity and significant changes of serum cytokine contents were found in GTKO mice, which perhaps suggested that the immune reactions to the DCM after 4 weeks of implantation were moderate and had minor effect on the survival of the corneal graft.

## Funding

This work was supported by the funds from National Key Research and Development Program of Ministry of science and technology of China (2016YFC1103200 and 2016YFC1103203).

*Conflict of interest statement*. The authors declare that they have no competing interests.
